# Study on the Influencing Factors of the Demand of Rural Older Adults in China for Elderly Care Services

**DOI:** 10.3390/healthcare13091086

**Published:** 2025-05-07

**Authors:** Linjing Wan, Xiaodong Di

**Affiliations:** School of Public Policy and Administration, Xi’an Jiaotong University, No 28 Xianning West Road, Xi’an 710049, China; wanlinjing@xjtu.edu.cn

**Keywords:** rural older adults, elderly care services, care needs, influencing factor, access to health

## Abstract

**Background/Objectives:** Population aging has become a common concern worldwide. At present, the aging rate of China far exceeds the international standard, and the rural population in China faces a more obvious aging problem. With the increasing number of the older population, the demand for elderly care services is constantly diversified, and the homogenized service supply in rural areas fails to effectively meet the service needs of older adults. **Methods:** This study employs a multi-stage stratified sampling method to survey rural older adults in Shaanxi, Hebei and Jiangsu provinces (n = 803, effective response rate > 95%). The dependent variable is categorized into four levels: no, mild, moderate and severe demands. Independent variables include demographic characteristics (age and gender), predisposing factors (education and marital status), enabling resources (income and family support) and need factors (health status). In the survey, the questionnaire survey method is adopted, and a multinomial logistic regression model is used to analyze the factors influencing the demand degree for elderly care services in rural areas. **Results:** Regression analysis indicates that pension level exerts a significant influence on the demand intensity for medical care, entertainment and spiritual comfort services. Family support is significantly associated with the demand intensity for medical care and spiritual comfort services. This study reveals that the pension level of elderly adults in rural areas is a key factor affecting the demand degree for elderly care services. The influence of family support on the demand for elderly care services should not be underestimated. Older adults in rural areas have a high demand for medical care services. **Conclusions:** A gap remains between elderly care service resources and the needs of older adults in rural areas of China. The government should pay attention to allocating and optimizing elderly care service resources to meet the needs of older adults.

## 1. Introduction

Population aging has become a social issue in the world. Currently, China is in a period of deepening population aging. According to the data of the National Bureau of Statistics, the population aged 60 and above has reached 280.04 million by the end of 2022 and accounts for 19.8% of the national population, of whom 209.78 million people are aged 65 and above and comprise 14.9% of the population [1]. It can be seen that the aging rate of China far exceeds the international standard, and the degree of aging is serious. In China, the aging problem of the rural population is more obvious than that of the urban population. Data from the seventh China Census show that the proportion of people over the age of 60 and 65 in rural areas is 23.81% and 17.72%, respectively, which is 7.99% and 6.61% higher than that in urban areas [2]. The intensification of the aging trend in rural areas has brought great challenges to the socioeconomic development of China, and great attention must be paid to the issue of rural elderly care [3]. Developing rural elderly care services is not only an inevitable requirement for the implementation of the rural revitalization strategy but also a key measure for the implementation of the national strategy to actively respond to the issue of population aging.

In the current situation of rural elderly care services in China, traditional family elderly care is faced with increasing pressure under the influence of family miniaturization and population outflow [4]. In addition, the labor cost of family care has increased dramatically with the increase in social competition, life pressure and work pressure [5]. These new changes have a huge impact on rural elderly care services, which results in the inability of rural older adults to receive enough family care. Additionally, market-oriented institutional elderly care services develop extremely slowly in rural areas, which is largely due to lower income levels and the influence of traditional beliefs among older adults. Therefore, the elderly care services provided by local communities and governments have become more critical [6]. Rural elderly care services are community services provided by local communities and municipalities. They also have increasingly incorporated the features of primary healthcare with the recent advancement of integrated care. Rural elderly care services encompass disability care, integrated care, meal assistance, visitation and emotional support and educational and recreational activities. Primarily focused on prevention, these services are gradually evolving into a comprehensive elderly care system integrating prevention, diagnosis, treatment, rehabilitation and palliative care. The financial funds for rural elderly care services are limited; the function of rural elderly care institutions is single; service quality is low, as service personnel are lacking in professional training; in most rural areas, specific public welfare organizations providing corresponding support for elderly care services are lacking [7]. With the increasing number of the older population, however, the homogenized service supply in rural areas fails to effectively meet the diversified service needs of older adults.

This paper uses the research data of the research group in Jiangsu, Hebei and Shaanxi provinces from 2020 and builds an analysis framework of influencing factors based on the Anderson service model. It analyzes the influencing factors of the demand for elderly care services in rural areas and puts forward countermeasures and suggestions to optimize the content of rural elderly care services according to the research results. The purpose is to accurately grasp the demand for and the influencing factors of elderly care services and to timely adjust the construction direction of elderly care services in rural areas.

## 2. Theory

In 1964, Anderson proposed the “Health Service Utilization Model”, which pointed out that individuals are affected by three dimensions—predisposing characteristics, enabling resources and need factors—when deciding whether to use health services [8]. Firstly, predisposing characteristics refer to the sociocultural tendency of individuals before they become ill or seek to use health services, and they have no direct relationship with the use of health services. They specifically include demographic characteristics (e.g., age and gender), social structural characteristics (e.g., individuals’ ethnicity, occupation, education, culture, social interaction and social networks) and health belief traits (e.g., individuals’ attitudes, values and perceptions of the health service system) [9]. Secondly, enabling resources refer to the capacity of individuals to obtain health services and the availability of health resources in communities and families. They are a factor that indirectly affects the utilization of health services, including economic status (e.g., income, medical insurance, marital status and the number of children) and community resources (e.g., the price of health services, the availability of community health resources, waiting time and consultation) [10]. Thirdly, needs refer to individual characteristics based on health needs. They represent individuals’ cognitive needs for health services (e.g., the subjective judgment of their own disease and health statuses) and assessment needs (e.g., the objective measurement and professional evaluation of patients’ health status by doctors) [11]. Needs are the most direct reason for the decision of individuals on whether to use health services. Cognitive needs mean that individuals can better understand seeking medical services and treatment options, and assessment needs are related to the type, quantity and quality of health services.

This paper focuses on the influencing factors of rural elderly adults’ demand for elderly care services. Therefore, predisposing characteristics, enabling resources and need factors under individual characteristics are examined, and external factors, namely situational characteristics, are introduced. The selection of factors influencing the demand for elderly care services depends not only on the individual function, living conditions and family resources of older adults but also on the contents of elderly care services, socioeconomic characteristics and other external factors [12]. Based on the full consideration of data availability and combining the development status of rural elderly care, this paper localizes the indicators affecting the demand for care services in Anderson’s model and summarizes the influencing factors of the demand for rural elderly care services into the following four categories.

Situational characteristic factors. Situational characteristics are mainly concerned with external environmental factors. For the construction and development of rural elderly care services, the supply of service content is naturally the most important external environmental factor [13]. Following the actual development status of rural elderly care services, the degree of social and market participation is unsatisfactory. Hence, in terms of situational characteristics, this paper mainly includes the service content provided by the government.

Predisposing characteristic factors. Demographic characteristics usually comprise gender, age and other factors, and social structure normally includes factors such as education level, marital status and living style [14]. This article takes the influencing factors of the demand for rural elderly care services as the research object. As a result, the influence of health belief on medical behavior in Anderson’s model is transformed into a discussion of the influence of self-care belief on elderly care demand.

Enabling resource factors. Enabling resources contain the resource elements possessed by older adults themselves and the resource elements that older adults can obtain from the outside world. Included in the variable system of Anderson’s model, “organization” represents access to health services, such as distance and time, the price of health services and social support [15]. According to the research focus of this paper, “organization” factors are refined into enabling resource factors.

Need factors. Need factors cover two dimensions of cognition and evaluation and refer to the subjective perception of individual health status and the objective measurement of actual illness [16]. This paper selects need factors according to Anderson’s model.

By clarifying the influencing factors of the demand for rural elderly services, this paper establishes an empirical model from four aspects—situational characteristics, predisposing characteristics, enabling resources and need factors—and analyzes the influencing mechanism of different factors on elderly care services.

## 3. Materials and Methods

### 3.1. Data Source

From December 2020 to March 2021, a questionnaire survey was conducted on “The Living Conditions and Development of Elderly Care Services in Rural Areas” in Shaanxi, Hebei and Jiangsu provinces. The research team consisted of more than 20 teachers, doctoral students and master’s students. All of the researchers received professional training and participated in pre-research. A multi-stage stratified sampling method was adopted in this survey. First, Jiangsu, Hebei and Shaanxi provinces were chosen as the first layer. Second, the method of stratified simple random sampling was employed to randomly select counties from cities as the second layer. Finally, convenient random sampling was used to select rural areas from each county as the third layer. The research group performed a survey on older adults aged 60 and above in 56 rural areas of the above-mentioned three provinces and collected 803 questionnaires. By sorting out questionnaires and eliminating the ones with many missing values and filling errors, 798 valid questionnaires were obtained, including 282 from Jiangsu, 233 from Hebei and 283 from Shaanxi, with an effective rate of more than 95%.

The questionnaire includes the needs of rural elderly care services and their influencing factors from different dimensions. Based on the four dimensions of Anderson’s model—situational characteristic, predisposing characteristic and enabling resource and need factors—this paper designs a questionnaire from the following four aspects by referring to the selection of factors for elderly care services by Wang et al. and Xing et al. [17,18]. The first part is the basic information about older adults, including gender, age, education level, marital status, the number of children, family income, etc. The second part mainly investigates the health status and life quality of older adults, including daily activity ability, illness, elderly care concept, etc. The third part involves investigating family supporting function and intergenerational relationships, including older adults’ living styles and relationships with their children. The fourth part is the demand for and utilization of elderly care services, including elderly service demand, service effect and suggestions, etc. Among them, the personal and family situations of older adults and the need for elderly care services are the key contents of the investigation. Considering the particularity of older adults, every two investigators formed a team in the actual research, with one being responsible for communicating with older adults and the other taking charge of recording.

### 3.2. Variable Selection

The dependent variable is the demand degree for elderly care services. The demand degree is specifically reflected in the number of demands for life care, medical care, entertainment and spiritual comfort services [19]. According to the number of demands, the demand degree is classified into four levels: no, mild, moderate and severe demands. The 5-point Likert scale is used as the method for assessing indicators, and values of “1, 2, 3, 4 and 5” are assigned according to the demand degree from weak to strong.

Independent variables are composed of predisposing characteristics, enabling resource and need factors and 10 indicators. The specific type, name and assignment of variables are shown in Table 1. Among them, service content completeness, neighborhood relationship, elderly care attitude, self-rated health status and self-rated psychological status are measured by the 5-point Likert scale. Age is grouped according to the actual survey data, and living style and chronic disease are treated as dummy variables. Pension level and family support as continuous variables are measured according to the actual survey data [20].

### 3.3. Research Method

This paper uses a multinomial logistic regression model to study the factors affecting the demand degree of older adults for elderly care services in rural areas. Multinomial logistic regression can be separated into ordered and disordered logistic regression [21]. Ordered logistic regression is characterized by a grade difference in dependent variables, such as no, mild, moderate and severe demands in this paper, which can be regarded as a kind of increasing demand level. Nevertheless, the differences in the dependent variables of disordered logistic regression belong to class differences, such as disease types. Hence, an ordered multinomial logistic regression model can be constructed.

(1) $$\mathrm{Logit}(p_{1})=\ln\frac{p\left(z_{0}\right)}{p\left(z_{3}\right)}=\alpha_{1}+\sum_{k=1}^{n}\beta_{1k}X_{k}+\varepsilon_{1}$$

(2) $$\mathrm{Logit}(p_{1}+p_{2})=\ln\frac{p\left(z_{1}\right)}{p\left(z_{3}\right)}=\alpha_{2}+\sum_{k=1}^{n}\beta_{2k}X_{k}+\varepsilon_{2}$$

(3) $$\mathrm{Logit}(p_{1}+p_{2}+p_{3})=\ln\frac{p\left(z_{2}\right)}{p\left(z_{3}\right)}=\alpha_{3}+\sum_{k=1}^{n}\beta_{3k}X_{k}+\varepsilon_{3}$$

In the equation, *p* denotes the demand of rural older adults for elderly care service projects; *Z*_0_, *Z*_1_ mild, *Z*_2_ and *Z*_3_ represent no, mild, moderate and severe demands, respectively; *α* stands for the constant term; *β_nk_* refers to the regression coefficient of the influencing factor; *X* indicates the independent variable; *ε* denotes the random error term.

## 4. Results

### 4.1. Basic Situation of Older Adults in Rural Areas

In terms of situational characteristics, the survey results show that most older adults give a relatively negative evaluation of service content completeness. Among them, 393 older adults hold that the service content provided by the village is extremely incomplete; 295 older adults think that it is not complete; 86.22% of older adults maintain that the service content is below the average level; only 4.51% of older adults consider the service content to be very complete.

Regarding predisposing characteristics, males and females account for 50.88% and 49.10%, respectively. In the aspect of age, the respondents comprise a large number of young older adults, among whom 389 are aged 60–69 and account for the largest proportion of 48.75%. The number of older adults aged 70–79 is 322 and accounts for 40.35%, while that of older adults aged 80 or over is relatively small. Concerning living conditions, 15.16% of older adults live alone; 65.16% live with their spouses; 46.12% live with their children; 15.79% live with their grandchildren; a very small number of older adults live with relatives and account for 1.38%. In terms of neighborhood relationships, over 80% of older adults believe that they have a good relationship with their neighbors, and only 0.88% think that they have a poor relationship with their neighbors. With regard to elderly care attitude, a majority of older adults agree that they are unable to completely rely on their children, accounting for 73.44%, but 15.41% of older adults disagree with this view.

From the perspective of enabling resources, in terms of pension level, the per capita income of rural older adults is generally low. Older adults with CNY 3000 or less account for 34.09%; about 7.77% of the respondents have no income; older adults with CNY 3001–6000 account for 20.93%, while those with CNY 6001 and above account for around 45%. Concerning family support, most children can provide limited financial support, and only 7.14% of children can provide older adults with financial support. However, children can provide more support concerning accompanying doctors, living care and daily concern, accounting for 20.93%, 22.93% and 23.56%, respectively.

From the angle of need factors, in terms of self-rated psychological status, the proportion of older adults with good and poor psychological statuses is more than 72% and approximately 12%, respectively. From the perspective of self-rated health status, more than 50% of older adults think that their health is relatively good, and only 3.63% consider their health to be very poor. In terms of chronic diseases, 22.93% of older adults have no diseases; more than 77% have at least one chronic disease; 38.10% suffer from cardiovascular and cerebrovascular diseases; and 24.31% develop bone and joint diseases. In addition, respiratory diseases, eye diseases, chronic gastroenteropathy and diabetes are also common chronic diseases among older adults.

### 4.2. Utilization of Services for Older Adults in Rural Areas

The survey results show that the most used service type for older adults is medical services, among which village clinics have the highest utilization rate. To be specific, 433 older people express that they go to village clinics to see a doctor, accounting for 54.26%. Health management has the second highest utilization rate. Specifically, 311 older people express that they have used the service, accounting for 38.97%. The third is the utilization rate of rehabilitation care is relatively low. Only nine people report that they have used rehabilitation care, accounting for 1.12%. In addition, another 11 older people report that they have used other medical services. Culture and entertainment rank second, and 42.98% of older adults, namely 343 people, suggest they have enjoyed leisure and entertainment services. The utilization rate of cultural activities is also relatively high with a total of 172 older people, accounting for 21.55%. The utilization rate of interest groups is low. Only 23 older people suggest that they have used the service, accounting for only 2.89%. The utilization rate of life care services is relatively low. Dining, cleaning assistance, security and home maintenance are used by 22, 45, 47 and 61 people, respectively, with no more than 200 people. The utilization rate of home maintenance is relatively high, whereas that of life care services is generally low, with less than 25% of older adults using the service. The utilization rate of life care services is even lower than that of some individual services like medical care and cultural entertainment. Spiritual comfort services have the lowest service utilization rate of below 20%, with fewer than 150 people. Psychological counseling, accompanying chat, reading newspapers and regular or telephone visits are utilized by 16, 25, 43 and 43 people, respectively, and the utilization rate of marriage intermediaries is zero. Another 17 older people report that they have used other spiritual comfort services.

### 4.3. Reliability and Validity Tests

In the questionnaire, only some of the items are measured by the scale, and most questionnaire items are randomly selected, without reliability and validity testing. Therefore, this paper tests the reliability and validity of only 23 items from five independent variables. In terms of the reliability test, Statistical Package for the Social Sciences (SPSS) 20 software is leveraged to measure the Alpha coefficient of the questionnaire. The larger the Alpha coefficient, the higher the reliability. The overall reliability analysis result of 23 items is 0.937. The results in Table 2 show that it has good consistency and stability and can be used for the following analysis.

In terms of the validity test, SPSS20 is used to conduct the Kaiser–Meyer–Olkin (KMO) and Bartlett sphericity tests on the research data, and the results are shown in Table 3. The results demonstrate that KMO = 0.894, and the significance of the Bartlett sphericity test is 0.000 < 0.01, which indicates good validity of the data.

### 4.4. Results of Influencing Factors of Demand Degree for Life Care Services

This paper constructs four models for the demand degree for four types of service contents, namely life care, medical care, entertainment and spiritual comfort services. All independent variables are included in the regression model. The collinear diagnosis of the four models shows that the tolerance is greater than 0.1, and the variance inflation factor is less than 10, thus indicating no multicollinearity.

Firstly, the test of parallel lines is carried out. The test results of the demand degree model of life care services are shown in Table 4, and *p* = 0.178 > 0.05, which indicates that the hypothesis of parallelism is valid. That is, all regression equations are parallel to each other, and ordered logistic regression can be used for analysis.

From the perspective of the goodness of model fit, the Pearson test result is *p* = 0.076 > 0.05, and the deviance test result is *p* = 1 > 0.05, which indicates that the model fit is good. From the perspective of the overall significance of the model, the likelihood ratio chi-square statistic of the model is 135.917, and the corresponding *p*-value is 0.000, less than 0.05, which indicates that the model is globally significant. The overall correct judgment ratio of the model exceeds 70%, and the specific regression analysis results are shown in Table 5.

### 4.5. Results of Influencing Factors of Demand Degree for Medical Care Services

In the test of parallel lines, χ^2^ = 8.620 and *p* = 0.000 < 0.05, which shows that the hypothesis of parallelism is not valid. That is to say, the regression equations are not parallel to each other, and the ordered logistic model cannot be used. Therefore, disordered multi-classification logistic regression is used to analyze the demand degree for medical care services (Table 6).

From the perspective of the overall significance of the model, the likelihood ratio chi-square statistic of the model is 334.793, and the corresponding *p*-value is 0.000 < 0.05, which indicates that the model is globally significant. In terms of model fit, the Cox–Snell and Nagelkerke values are 0.428 and 0.470, respectively. The overall correct judgment ratio of the model is 57.3% (Table 7).

### 4.6. Results of Influencing Factors of Demand Degree for Entertainment Services

First, the test of parallel lines is conducted. χ^2^ = 90.364 and *p* = 0.963 > 0.05, which indicates that the hypothesis of parallelism is valid. In other words, all regression equations are parallel to each other, and the ordered logistic process can be used for analysis (Table 8).

The results of the Pearson (*p* = 0.196 > 0.05) and deviance tests (*p* = 1 > 0.05) show that the model is well fitted. The likelihood ratio chi-square statistic of the model is 154.233, and the corresponding *p*-value is 0.000 < 0.05, which indicates that the model is significant as a whole. The overall correct judgment rate of the model is 47.7% (Table 9).

### 4.7. Results of Influencing Factors of Demand Degree for Spiritual Comfort Services

First, the test of parallel lines is carried out. χ^2^ = 129.933 and *p* = 1 > 0.05 indicate that the hypothesis of parallelism is valid. That is, the regression equations are parallel to each other, and the ordered logistic process can be used for analysis (Table 10).

The result of the Pearson test (*p* = 0 < 0.05) indicates that the model is poorly fitted, while that of the deviance test (*p* = 1 > 0.05) indicates that the model is well fitted. The likelihood ratio chi-square statistic of the model is 159.958, and the corresponding *p*-value is 0.000 < 0.05, which indicates that the model is significant as a whole. The overall correct judgment ratio of the model is 76.8% (Table 11).

## 5. Discussion

### 5.1. Analysis of Life Care Services

In terms of situational characteristics, service content completeness exerts an important impact on the demand degree for life care services. The results show that the more complete the service content provided by the government or village collective is, the greater the demand for elderly care services will be. From the perspective of occurrence ratio, service content completeness decreases by one level, and the demand of older adults for life care services decreases by more than 50%. The demand degree of areas with extremely incomplete service content is 0.079 times that of areas with complete service content and significant at the 1% level, which well reflects that the subjective needs of older adults are greatly affected by objective conditions. Understandably, if the service content provided is low, older adults will naturally reduce their own needs under limited conditions. Conversely, rich service content will increase and stimulate the demand for elderly care services [22].

In terms of predisposing characteristics, the living style and elderly care attitude of older adults pass the significance test at the level of 10%. From the perspective of living style, older adults living alone show a higher demand degree for elderly care services because they are in a more vulnerable state and usually unable to accomplish numerous things by themselves [23]. In contrast, older adults living with their grandchildren show a lower demand degree for elderly care services, decreasing by nearly 20%. Elderly adults willing to participate in elderly care services show a higher demand degree for life care services than those unwilling to participate in elderly care services, with an increase of more than 60%. Older adults willing to participate in elderly care services rely more on their children or the government, show higher dependence on others and hope to get more help from the outside world.

In terms of enabling resources, family support passes the significance test, which indicates that family support has an impact on the life care needs of older adults. The survey results reveal that older adults who receive medical support have the highest demand for life care, and those who receive daily concern have the lowest demand. The former is 1.896 times that of the latter. This result reflects that older adults receiving medical support have poorer physical conditions, are less able to cope with daily tasks and require more life support, which results in an increased need for corresponding care services. Thus, the government needs to take “family policy” as the starting point and emphasize the integration of different elderly care service models in the process of promoting the development of rural elderly care services.

In terms of need factors, self-rated health status and chronic disease pass the significance test. Older adults with good self-rated health status show a lower demand for elderly care services, and older adults with chronic diseases show a higher demand. This result reflects the inverse relationship between the physical status of older adults and the demand degree for elderly care services [24]. With the aging of the body, older adults will develop more diseases, and their mobility will also be greatly affected by their physical function.

### 5.2. Analysis of Medical Care Services

In terms of situational characteristics, service content completeness passes the significance test. From the perspective of occurrence ratio, the probability of older adults who think that “service content is very incomplete” with moderate medical care service needs is 0.023 times that of those who think that “service content is very complete”. It is significant at the level of 1%, which is consistent with the above discussion. The more complete medical care services are, the more the demand of older adults for corresponding services can be improved. This is in line with the actual situation of elderly care services in central and western China and shows a lower level of elderly care service construction and supply capacity.

In terms of predisposing characteristics, age and elderly care attitude pass the significance test, which indicates that the two variables have an important impact on the demand degree for medical care services. From the perspective of age, the demand of older adults for moderate and severe medical care services also increases correspondingly with the increase in age and is significant at the level of 1%. The higher the age, the more serious the physical degradation, and the demand for medical care services also increases correspondingly [25]. From the angle of elderly care attitude, older adults willing to participate in elderly care services have a higher demand for medical care. This may be because older adults with a positive attitude show a higher willingness to use social services to reduce the pressure of family care.

In terms of enabling resources, pension level and family support pass the significance test. The probability of older adults who have a pension level of CNY 0–3000 and show a moderate demand for medical care services is over 60% higher than that of those with a pension level of CNY 6001–15,000. In terms of family support, the probability of older adults who receive financial support and have a moderate demand for medical care services is 2.659 times that of those who receive daily concern. It can be seen that the economic ability of older adults has a very significant impact on their demand for medical care services [26], which is aligned with the actual situation. Compared with other types of services, medical care services such as drug purchase, nursing and rehabilitation care typically require more financial support. As a result, older adults at a lower economic level show a higher demand for medical care services.

In terms of need factors, self-rated psychological and health statuses have a certain impact on the demand degree for elderly care services. From the perspective of occurrence ratio, the probability of older adults who have “very poor self-rated psychological status” and show a moderate demand for medical care services is 4.820 times that of those who have “very good self-rated psychological status” and is significant at the level of 10%. The probability of older adults who have “good self-rated health” and show a severe demand for medical care is slightly higher than that of those who have “poor self-rated health”, which is inconsistent with the hypothesis. In addition, chronic diseases do not pass the significance test, which is also different from the expected results.

### 5.3. Analysis of Entertainment Services

In terms of situational characteristics, service content completeness still passes the significance test and is significant at the 1% level. From the perspective of occurrence ratio, the service demand of older adults who think that elderly care services are incomplete is 0.091 times that of those who think that elderly care services are very complete, with a decrease of more than 90%. This result reflects the relative lack of entertainment services in rural areas and the relatively simple entertainment ways of rural older adults.

In terms of predisposing characteristics, neighborhood relationships pass the significance test, which is consistent with the actual situation. In most cases, interest groups for older adults or some cultural activities are an interaction between older adults, which requires the support of good neighborhood relations [27]. The demand degree for entertainment of older adults who think that neighborhood relationships are normal is 0.775 times that of those who think that neighborhood relationships are very good. This indicates that the demand degree for entertainment also changes with the change in the relationship between older adults and the neighborhood and shows a positive correlation trend.

In terms of enabling resources, the pension level passes the significance test. Most rural older adults are busy with farm work and other affairs and spend most of their time making a living. Older adults with a higher level of pension face relatively small livelihood pressure and have more time and energy to take part in entertainment activities [28]. Older adults with a pension level of CNY 10,001–15,000 show the highest demand for entertainment services, which is 200% higher than that of those with other pension levels. This is a good demonstration of enjoying old age with money.

In terms of need factors, self-rated psychological status passes the significance test and exerts a positive effect on the demand degree for entertainment services. Only in a good psychological state are older adults more willing to invest in recreational activities. In addition, a good psychological state can also help older adults better integrate into various recreational activities, which in turn increases their corresponding demand degree [29].

### 5.4. Analysis of Spiritual Comfort Services

In terms of situational characteristics, service content completeness passes the significance test, which reflects that the corresponding demand of older adults also increases with the improvement in service content completeness. By comparing the data of the four types of services, it can be found that the demand of rural older adults for spiritual comfort services is generally at a relatively low level, which is related to the fact that rural older adults mostly have little idea of spiritual comfort services [30].

In terms of predisposing characteristics, age and living style pass the significance test. From the perspective of age, older adults aged 60–79 have a higher demand degree for spiritual comfort services, which is significant at the level of 1%. Older adults over 80 years old have a relatively low demand degree for spiritual comfort services. From the perspective of living style, older adults living alone show a higher demand degree than those living with their spouses or children. Older adults living alone are lonelier, often deal with all affairs alone and have no way to express their inner emotions [31]. Therefore, they need more spiritual comfort services to relieve their troubles and depression.

In terms of enabling resources, pension level and family support pass the significance test. Older adults with a pension level of CNY 0–3000 show the strongest demand for services, while those with a pension level of CNY 10,001–15,000 have a lower demand for spiritual comfort services. From the perspective of occurrence ratio, the demand of the former for services increases by more than 120% compared with that of the latter. This result reflects the greater psychological pressure on older adults who are financially strapped [32]. From the perspective of family support, older adults receiving medical support show a higher demand degree. These older adults have relatively poor physical function and need to obtain more psychological relief and other related services. Thus, the government should attach importance to the coordinated development of regions and focus on narrowing inter-regional differences. It is advised to strengthen the financial, human and policy support for the construction of elderly care services in economically backward rural areas, optimize the allocation of resources in a variety of regions and increase the income of low-income older groups in rural areas.

In terms of need factors, self-rated health status and chronic disease have a negative impact on the demand degree for services [33]. For each grade of decrease in self-rated health status, the service demand degree of older adults increases by one grade. In terms of occurrence ratio, older adults with very poor self-rated health have 2.016 times higher demand for services than those with very good self-rated health. From the perspective of chronic disease, the service demand of older adults with five chronic diseases and above increases by more than 80% compared with that of older adults with no disease.

## 6. Conclusions

This paper takes rural older adults as the research object and carries out field investigations in Jiangsu, Hebei and Shaanxi provinces. Based on Anderson’s model, elderly care services are divided into four categories: life care, medical care, entertainment and spiritual comfort services. In addition, this paper analyzes the factors affecting the demand degree for elderly care services. The research conclusions are as follows. Firstly, the pension level of rural older adults is one of the key factors affecting the demand degree for elderly care services, which reflects the common sense that the economy is the basis of human survival and development. Secondly, in the rural society of China, family support plays an essential role in elderly care services. Thirdly, older adults in rural areas have a high demand for medical care services.

Since the survey data used in this paper are not tracking data, this paper fails to pay attention to changes in the demand degree of rural older adults for elderly care services in different periods. Moreover, the epidemic situation is still raging during the survey period. The demand of older adults may be in a transient unstable state, and the data obtained in the survey fail to represent their long-term demand.

Despite little difference in the number of districts, counties and villages selected in the three provinces, the number of cities selected in Jiangsu and Shaanxi provinces is significantly greater than that in Hebei province, and the representation of samples is poor. Future investigations can be conducted in more representative cities and provinces to obtain as many samples as possible and minimize the skewed distribution of research data.

In this paper, an insufficient number of relevant indicators of situational characteristics is included, and whether the demand of rural older adults for elderly care services is affected by informal factors is not taken into consideration. Moreover, the interaction between variables in the model is not explored in depth. It is necessary to verify and discuss more study variables and their interactions in future studies.

## Figures and Tables

**Table 1:** Variables, indicators and options.

Variables		Indicators	Options
Dependent variable		Demand degree for life care services	0. No demand 1. Mild demand 2. Moderate demand 3. Severe demand
		Demand degree for medical care	
		Demand degree for entertainment services	
		Demand degree for spiritual comfort services	
Independent variable	Situational characteristic	Service content completeness	1. Very incomplete 2. Not complete 3. Generally complete 4. Relatively complete 5. Very complete
	Predisposing characteristic	Age	1. 60~69 2. 70~79 3. 80~89 4. Above 90
		Living style	1. Living alone 2. Spouse 3. Children 4. Grandchildren 5. Relatives
		Neighborhood relationship	1. Bad 2. Average 3. Better 4. Well
		Elderly care attitude	1. Strongly disagree 2. Disagree 3. Generally agree 4. Agree 5. Strongly agree
	Enabling resource	Pension level	1. 0~3000 2. 3001~6000 3. 6001~10,000 4. 10,001~15,000 5. Above 15,000
		Family support	0. No support 1. Financial support 2. Medical support 3. Living care 4. Daily concern
	Need factors	Self-rated psychological status	1. Very bad 2. Bad 3. Average 4. Better 5. Well
		Self-rated health status	1. Very bad 2. Bad 3. Average 4. Better 5. Well
		Chronic disease	1. No disease 2. Have 1–4 chronic diseases 3. Have more than 5 chronic diseases

**Table 2:** Reliability analysis.

Variables	Number of Items	Cronbach’s Alpha
Service content completeness	4	0.742
Neighborhood relationship	3	0.903
Elderly care attitude	7	0.738
Self-rated health status	5	0.852
Self-rated psychological status	4	0.874

**Table 3:** Validity analysis.

KMO and Bartlett Tests		
KMO sample appropriateness measure		0.894
Bartlett sphericity test	Approximate chi-square	16,175.610
	df	2346
	Sig.	0.000

**Table 4:** Test of parallel lines.

Test of Parallel Lines				
Model	−2 Log Likelihood	Chi-Square	df	Sig.
Null hypothesis	1090.451			
General	960.518	129.933	116	0.178

Note: The null hypothesis states that the location parameters (slope coefficients) are the same across the response categories (same below).

**Table 5:** Results of regression analysis of the demand degree for life care services.

Variables		B	Std. E	Exp (B)	95% CI	
					Lower Limit	Upper Limit
Dependent variable	No demand	12.115 ***	2.003	9.97 × 10^6^	8.189	16.041
	Mild demand	13.146 ***	1.999	2.79 × 10^7^	9.227	17.065
	Moderate demand	14.360 ***	1.998	9.41 × 10^7^	10.445	18.275
Situational characteristic	[Service content completeness = 1]	−2.544 ***	0.745	0.079	−4.003	−1.085
	[Service content completeness = 2]	−1.412 *	0.743	0.244	−2.868	0.044
	[Service content completeness = 3]	−0.734	0.764	0.480	−2.232	0.764
	[Service content completeness = 4]	−1.040	0.822	0.353	−2.651	0.571
	[Service content completeness = 5]	0 ^a^		1		
Predisposing characteristic	[Age = 1]	−0.041	1.226	0.959	−2.444	2.361
	[Age = 2]	0.112	1.218	1.119	−2.276	2.501
	[Age = 3]	0.729	1.224	2.072	−1.670	3.127
	[Age = 4]	0 ^a^		1		
	[Living style = 1]	−1.338	0.869	0.262	−3.041	0.364
	[Living style = 2]	−1.589 *	0.834	0.204	−3.223	0.046
	[Living style = 3]	−1.547 *	0.862	0.213	−3.236	0.142
	[Living style = 4]	−2.573 *	1.414	0.076	−5.345	0.200
	[Living style = 5]	0 ^a^		1		
	[Neighborhood relationship = 1]	1.413	1.207	4.108	−0.953	3.779
	[Neighborhood relationship = 2]	0.156	0.276	1.169	−0.385	0.698
	[Neighborhood relationship = 3]	−0.118	0.225	0.889	−0.558	0.322
	[Neighborhood relationship = 4]	0 ^a^		1		
	[Elderly care attitude = 1]	0.507	0.449	1.660	−0.373	1.387
	[Elderly care attitude = 2]	0.054	0.334	1.056	−0.601	0.709
	[Elderly care attitude = 3]	−0.678 *	0.357	0.508	−1.378	0.023
	[Elderly care attitude = 4]	−0.115	0.241	0.892	−0.587	0.358
	[Elderly care attitude = 5]	0 ^a^		1		
Enabling resource	[Pension level = 1]	0.278	0.402	1.321	−0.509	1.065
	[Pension level = 2]	0.546	0.467	1.726	−0.370	1.461
	[Pension level = 3]	0.816	0.529	2.262	−0.221	1.854
	[Pension level = 4]	0.311	0.546	1.365	−0.758	1.381
	[Pension level = 5]	0 ^a^		1		
	[Family support = 0]	−1.186	1.453	0.305	−4.034	1.663
	[Family support = 1]	0.395	0.348	1.484	−0.286	1.077
	[Family support = 2]	0.640 ***	0.243	1.896	0.163	1.117
	[Family support = 3]	0.126	0.253	1.135	−0.370	0.623
	[Family support = 4]	0 ^a^		1		
Need factors	[Self-rated psychological status = 1]	0.461	0.514	1.585	−0.546	1.468
	[Self-rated psychological status = 2]	0.166	0.393	1.181	−0.603	0.936
	[Self-rated psychological status = 3]	0.203	0.276	1.226	−0.338	0.745
	[Self-rated psychological status = 4]	0.381	0.233	1.463	−0.077	0.838
	[Self-rated psychological status = 5]	0 ^a^		1		
	[Self-rated health status = 1]	−0.096	0.532	0.908	−1.139	0.947
	[Self-rated health status = 2]	−0.388	0.357	0.678	−1.087	0.311
	[Self-rated health status = 3]	−0.135	0.324	0.873	−0.770	0.500
	[Self-rated health status = 4]	−0.758 **	0.302	0.468	−1.351	−0.165
	[Self-rated health status = 5]	0 ^a^		1		
	[Chronic disease = 1]	−1.241	0.770	0.289	−2.750	0.267
	[Chronic disease = 2]	−1.474 **	0.747	0.229	−2.938	−0.009
	[Chronic disease = 3]	0 ^a^		1		

^a^: This parameter is redundant and therefore set to zero (same as below). Note: *** means significant at the 1% level, ** means significant at the 5% level, and * means significant at the 10% level. In particular, SPSS takes the severe demand of the dependent variable as the reference when carrying out ordered logistic regression. Thus, the interpretation of the odds ratio (OR) value is slightly special (same as below), and the analysis module cannot directly give the OR value. Hence, it needs to be calculated separately.

**Table 6:** Test of parallel lines.

Test of Parallel Lines				
Model	−2 Log Likelihood	Chi-Square	df	Sig.
Null hypothesis	1200.783			
General	944.844 ^b^	255.939 ^c^	116	0.000

Note: ^b^ means reaching the maximum number of stepwise bisections prevents further increases in the log-likelihood value. ^c^ means the chi-square statistic is calculated based on the log-likelihood value obtained from the final iteration of the general model.

**Table 7:** Results of regression analysis of the demand degree for medical care services.

Variables		B	Std. E	Exp (B)	95% CI	
					Lower Limit	Upper Limit
Dependent variable	No demand	8.100	6.061			
	Mild demand	18.066 ***	6.233			
	Moderate demand	45.428 ***	12.161			
Situational characteristic	[Service content completeness = 1]	−3.778 ***	1.301	0.023	0.002	0.293
	[Service content completeness = 2]	−1.894	1.306	0.150	0.012	1.946
	[Service content completeness = 3]	−0.972	1.386	0.378	0.025	5.728
	[Service content completeness = 4]	−1.218	1.535	0.296	0.015	5.992
	[Service content completeness = 5]	0 ^a^				
Predisposing characteristic	[Age = 1]	−12.335 *** −41.114 *** (Severe)	0.534 0.831	4.397 × 10^−6^ 1.395 × 10^−18^	1.544 × 10^−6^ 2.738 × 10^−19^	1.253 × 10^−5^ 7.104 × 10^−18^
	[Age = 2]	−12.083 *** −41.492 *** (Severe)	0.492 0.787	5.655 × 10^−6^ 9.552 × 10^−19^	2.157 × 10^−6^ 2.043 × 10^−19^	1.483 × 10^−5^ 4.465 × 10^−18^
	[Age = 3]	−12.059	0.000	5.793 × 10^−6^	5.793 × 10^−6^	5.793 × 10^−6^
	[Age = 4]	0 ^a^				
	[Living style = 1]	−1.127	4.048	0.324	0.000	904.467
	[Living style = 2]	−1.112	4.035	0.329	0.000	893.869
	[Living style = 3]	−0.676	4.045	0.509	0.000	1412.255
	[Living style = 4]	−0.791	4.163	0.453	0.000	1584.330
	[Living style = 5]	0 ^a^				
	[Neighborhood relationship = 1]	0.434	2.985	1.544	0.004	536.213
	[Neighborhood relationship = 2]	0.374	0.417	1.454	0.641	3.295
	[Neighborhood relationship = 3]	0.063	0.325	1.065	0.564	2.013
	[Neighborhood relationship = 4]	0 ^a^				
	[Elderly care attitude = 1]	0.080	0.857	1.083	0.202	5.814
	[Elderly care attitude = 2]	−1.268 *	0.506	0.281	0.104	0.758
	[Elderly care attitude = 3]	−0.658	0.494	0.518	0.197	1.365
	[Elderly care attitude = 4]	−0.441	0.360	0.643	0.317	1.303
	[Elderly care attitude = 5]	0 ^a^				
Enabling resource	[Pension level = 1]	1.536 **	0.608	4.645	1.410	15.304
	[Pension level = 2]	0.859 2.330 * (Severe)	0.754 1.308	2.361 10.280	0.538 0.792	10.352 133.515
	[Pension level = 3]	1.366 *	0.785	3.922	0.841	18.279
	[Pension level = 4]	1.395 *	0.787	4.033	0.863	18.843
	[Pension level = 5]	0 ^a^				
	[Family support = 0]	0.888	1.915	2.431	0.057	103.800
	[Family support = 1]	0.978 *	0.558	2.659	0.891	7.936
	[Family support = 2]	−0.021	0.353	0.979	0.490	1.957
	[Family support = 3]	−0.036	0.357	0.964	0.479	1.941
	[Family support = 4]	0 ^a^				
Need factors	[Self-rated psychological status = 1]	1.573 * (mild) 1.174	0.942 0.962	4.820 3.236	0.760 0.491	30.551 21.308
	[Self-rated psychological status = 2]	0.728	0.647	2.071	0.582	7.367
	[Self-rated psychological status = 3]	0.005	0.392	1.005	0.466	2.167
	[Self-rated psychological status = 4]	0.389	0.351	1.475	0.741	2.938
	[Self-rated psychological status = 5]	0 ^a^				
	[Self-rated health status = 1]	−1.284	0.914	0.277	0.046	1.660
	[Self-rated health status = 2]	−0.102 −1.954 * (Severe)	0.567 1.003	0.903 0.142	0.297 0.020	2.746 1.011
	[Self-rated health status = 3]	−0.122	0.508	0.885	0.327	2.394
	[Self-rated health status = 4]	−0.336 −1.571 ** (Severe)	0.465 0.709	0.714 0.208	0.287 0.052	1.776 0.834
	[Self-rated health status = 5]	0 ^a^				
	[Chronic disease = 1]	−1.151	1.385	0.316	0.021	4.779
	[Chronic disease = 2]	−0.421	1.356	0.656	0.046	9.366
	[Chronic disease = 3]	0 ^a^				

^a^: This parameter is redundant and therefore set to zero. Note: *** means significant at the 1% level, ** means significant at the 5% level, and * means significant at the 10% level. In particular, to optimize the presentation of the results, the main part of the table presented is “moderate demand” results, while “mild and severe demands” only show variables passing the significance test (same below).

**Table 8:** Test of parallel lines.

Test of Parallel Lines				
Model	−2 Log Likelihood	Chi-Square	df	Sig.
Null hypothesis	1321.196			
General	1230.833 ^b^	90.364 ^c^	116	0.963

Note: ^b^ means reaching the maximum number of stepwise bisections prevents further increases in the log-likelihood value. ^c^ means the chi-square statistic is calculated based on the log-likelihood value obtained from the final iteration of the general model.

**Table 9:** Results of regression analysis of the demand degree for entertainment services.

Variables		B	Std. E	Exp (B)	95% CI	
					Lower Limit	Upper Limit
Dependent variable	No demand	1.611	2.481	5.009	−3.252	6.475
	Mild demand	3.347	2.485	28.407	−1.524	8.217
	Moderate demand	5.171 **	2.488	176.149	0.294	10.048
Situational characteristic	[Service content completeness = 1]	−3.507 ***	0.742	0.030	−4.963	−2.052
	[Service content completeness = 2]	−2.392 ***	0.740	0.091	−3.842	−0.941
	[Service content completeness = 3]	−2.155 ***	0.764	0.116	−3.652	−0.658
	[Service content completeness = 4]	−1.251	0.811	0.286	−2.840	0.338
	[Service content completeness = 5]	0 ^a^		1		
Predisposing characteristic	[Age = 1]	2.007	1.450	7.440	−0.835	4.848
	[Age = 2]	1.635	1.446	5.131	−1.198	4.469
	[Age = 3]	1.006	1.457	2.735	−1.849	3.861
	[Age = 4]	0 ^a^		1		
	[Living style = 1]	0.538	0.861	1.712	−1.149	2.225
	[Living style = 2]	0.364	0.830	1.439	−1.263	1.991
	[Living style = 3]	0.598	0.853	1.818	−1.074	2.269
	[Living style = 4]	0.827	1.118	2.287	−1.364	3.019
	[Living style = 5]	0 ^a^		1		
	[Neighborhood relationship = 1]	−21.057	0.000	2.636 × 10^−10^	−21.057	−21.057
	[Neighborhood relationship = 2]	−0.255	0.245	0.775	−0.734	0.225
	[Neighborhood relationship = 3]	−0.437 **	0.193	0.646	−0.816	−0.059
	[Neighborhood relationship = 4]	0 ^a^		1		
	[Elderly care attitude = 1]	0.416	0.441	1.516	−0.448	1.280
	[Elderly care attitude = 2]	0.002	0.296	1.002	−0.577	0.581
	[Elderly care attitude = 3]	−0.322	0.298	0.725	−0.906	0.262
	[Elderly care attitude = 4]	0.101	0.213	1.106	−0.316	0.519
	[Elderly care attitude = 5]	0 ^a^		1		
Enabling resource	[Pension level = 1]	0.195	0.339	1.215	−0.469	0.859
	[Pension level = 2]	−0.373	0.421	0.689	−1.198	0.452
	[Pension level = 3]	0.409	0.468	1.506	−0.509	1.327
	[Pension level = 4]	1.267 ***	0.463	3.551	0.360	2.175
	[Pension level = 5]	0 ^a^		1		
	[Family support = 0]	0.711	0.889	2.037	−1.032	2.455
	[Family support = 1]	−0.197	0.313	0.821	−0.811	0.417
	[Family support = 2]	0.241	0.212	1.272	−0.175	0.657
	[Family support = 3]	−0.013	0.215	0.987	−0.435	0.408
	[Family support = 4]	0 ^a^		1		
Need factors	[Self-rated psychological status = 1]	−0.059	0.492	0.943	−1.022	0.905
	[Self-rated psychological status = 2]	0.386	0.341	1.472	−0.282	1.055
	[Self-rated psychological status = 3]	0.275	0.241	1.317	−0.197	0.748
	[Self-rated psychological status = 4]	0.421 **	0.203	1.524	0.023	0.820
	[Self-rated psychological status = 5]	0 ^a^		1		
	[Self-rated health status = 1]	−0.403	0.513	0.668	−1.409	0.602
	[Self-rated health status = 2]	0.143	0.317	1.154	−0.479	0.765
	[Self-rated health status = 3]	−0.328	0.291	0.720	−0.899	0.243
	[Self-rated health status = 4]	0.066	0.262	1.069	−0.447	0.579
	[Self-rated health status = 5]	0 ^a^		1		
	[Chronic disease = 1]	0.774	0.808	2.169	−0.809	2.357
	[Chronic disease = 2]	0.926	0.790	2.524	−0.622	2.474
	[Chronic disease = 3]	0 ^a^		1		

^a^: This parameter is redundant and therefore set to zero. Note: *** means significant at the 1% level and ** means significant at the 5% level.

**Table 10:** Test of parallel lines.

Test of Parallel Lines				
Model	−2 Log Likelihood	Chi-Square	df	Sig.
Null hypothesis	740.560			
General	687.219 ^b^	53.341 ^c^	116	1.000

Note: ^b^ means reaching the maximum number of stepwise bisections prevents further increases in the log-likelihood value. ^c^ means the chi-square statistic is calculated based on the log-likelihood value obtained from the final iteration of the general model.

**Table 11:** Results of regression analysis of the demand degree for spiritual comfort services.

Variables		B	Std. E	Exp (B)	95% CI	
					Lower Limit	Upper Limit
Dependent variable	No demand	25.731 ***	1.767	4.46 × 10^14^	22.267	29.196
	Mild demand	27.986 ***	1.750	4.25 × 10^15^	24.557	31.415
	Moderate demand	29.878 ***	1.779	2.82 × 10^16^	26.391	33.364
Situational characteristic	[Service content completeness = 1]	−3.740 ***	0.807	0.024	−5.322	−2.158
	[Service content completeness = 2]	−2.446 ***	0.796	0.087	−4.006	−0.886
	[Service content completeness = 3]	−1.108	0.808	0.330	−2.693	0.476
	[Service content completeness = 4]	−1.543 *	0.867	0.214	−3.243	0.157
	[Service content completeness = 5]	0 ^a^		1		
Predisposing characteristic	[Age = 1]	16.399 ***	0.375	7.23 × 10^8^	15.664	17.135
	[Age = 2]	16.517 ***	0.352	8.13 × 10^8^	15.826	17.207
	[Age = 3]	17.048	0.000	1.38 × 10^9^	17.048	17.048
	[Age = 4]	0 ^a^		1		
	[Living style = 1]	−1.603 *	0.914	0.201	−3.395	0.189
	[Living style = 2]	−1.808 **	0.873	0.164	−3.519	−0.098
	[Living style = 3]	−2.084 **	0.915	0.124	−3.878	−0.290
	[Living style = 4]	−1.015	1.262	0.362	−3.487	1.458
	[Living style = 5]	0 ^a^		1		
	[Neighborhood relationship = 1]	−1.404	1.502	0.246	−4.348	1.541
	[Neighborhood relationship = 2]	0.031	0.319	1.031	−0.594	0.656
	[Neighborhood relationship = 3]	−0.167	0.260	0.847	−0.677	0.343
	[Neighborhood relationship = 4]	0 ^a^		1		
	[Elderly care attitude = 1]	−1.538	0.689	0.215	−2.889	−0.188
	[Elderly care attitude = 2]	−0.220	0.396	0.803	−0.997	0.557
	[Elderly care attitude = 3]	−0.426	0.390	0.653	−1.190	0.338
	[Elderly care attitude = 4]	0.100	0.274	1.105	−0.437	0.637
	[Elderly care attitude = 5]	0 ^a^		1		
Enabling resource	[Pension level = 1]	0.384	0.440	1.468	−0.478	1.246
	[Pension level = 2]	−0.059	0.536	0.943	−1.110	0.992
	[Pension level = 3]	0.275	0.600	1.317	−0.900	1.450
	[Pension level = 4]	−1.416 *	0.816	0.243	−3.016	0.184
	[Pension level = 5]	0 ^a^		1		
	[Family support = 0]	−1.443	1.817	0.236	−5.004	2.118
	[Family support = 1]	0.052	0.408	1.053	−0.748	0.852
	[Family support = 2]	0.621 **	0.275	1.861	0.081	1.161
	[Family support = 3]	0.202	0.291	1.224	−0.369	0.773
	[Family support = 4]	0 ^a^		1		
Need factors	[Self-rated psychological status = 1]	−0.471	0.620	0.625	−1.686	0.744
	[Self-rated psychological status = 2]	−0.513	0.465	0.599	−1.424	0.398
	[Self-rated psychological status = 3]	−0.237	0.324	0.789	−0.872	0.398
	[Self-rated psychological status = 4]	−0.139	0.269	0.870	−0.666	0.388
	[Self-rated psychological status = 5]	0 ^a^		1		
	[Self-rated health status = 1]	0.701	0.604	2.016	−0.482	1.884
	[Self-rated health status = 2]	0.017	0.399	1.018	−0.765	0.800
	[Self-rated health status = 3]	−0.031	0.369	0.969	−0.755	0.693
	[Self-rated health status = 4]	−0.800 **	0.347	0.449	−1.480	−0.121
	[Self-rated health status = 5]	0 ^a^		1		
	[Chronic disease = 1]	−1.693 **	0.853	0.184	−3.365	−0.021
	[Chronic disease = 2]	−1.436 *	0.822	0.238	−3.047	0.175
	[Chronic disease = 3]	0 ^a^		1		

^a^: This parameter is redundant and therefore set to zero. Note: *** means significant at the 1% level, ** means significant at the 5% level, and * means significant at the 10% level.

## Data Availability

Data are contained within the article.
